# Kinetic Analysis and Products Characterization of Hydrothermal Liquefaction of Tetra Pak Waste for Bio-Oil Production

**DOI:** 10.3390/polym17243246

**Published:** 2025-12-05

**Authors:** Yuzhen Wang, Ao Lu, Zhuan Liu, Yu Feng, Di Shan, Changqing Fang

**Affiliations:** Faculty of Printing, Packaging Engineering and Digital Media Technology, Xi’an University of Technology, Xi’an 710048, China

**Keywords:** hydrothermal liquefaction, Tetra Pak, bio-oil, kinetics, activation energy

## Abstract

Hydrothermal liquefaction (HTL) of Tetra Pak waste was investigated at 320–440 °C for 10–50 min to produce bio-crude oil. Bio-oil yield increased with temperature and time, reaching about 43 wt% at 40–50 min, while solid residue decreased and stabilized. Boiling point analysis indicated diesel- and kerosene-range fractions as dominant components. FT-IR results showed enhanced aromatic and carbonyl groups with reaction time, suggesting secondary condensation. A modified first-order kinetic model described the conversion of carbohydrates and polyethylene, with activation energies of 25.8–49.0 and 54.9–78.3 kJ mol^−1^, respectively. The intermediate aqueous/gaseous pathway exhibited a lower activation energy (30.1 kJ mol^−1^), highlighting its vital role in oil formation. This study advances understanding of Tetra Pak liquefaction and provides guidance for efficient composite waste valorization.

## 1. Introduction

The increasing accumulation of packaging waste poses a significant environmental challenge worldwide. Among these, Tetra Pak cartons—widely used for liquid food packaging—are particularly difficult to recycle due to their composite structure of paper, plastic, and aluminum layers [1]. Globally, more than 190 billion Tetra Pak cartons are produced annually, generating over 1.2–1.4 million tons of post-consumer waste each year. However, the global recycling rate remains only 26%, leaving the majority landfilled or incinerated [2]. In China alone, annual production exceeds 30 billion cartons, corresponding to more than 200,000 tons/year of waste, but the effective recycling rate is reported to be below 20% because of the difficulty in separating the paper–plastic–aluminum layers [3]. These data highlight the urgent need for alternative valorization technologies for multilayer packaging waste. Conventional end-of-life treatments such as landfilling and incineration remain dominant but result in resource loss and potential secondary pollution. Thermal conversion routes, including pyrolysis and gasification, have also been investigated for composite or polymeric wastes; however, these processes often lead to high char formation from cellulose, incomplete aluminum separation, and quality deterioration when mixed organic–inorganic materials are treated simultaneously. These limitations underline the need for an alternative pathway capable of deconstructing such multi-layered composites while recovering energy in a more controlled manner.

In response to these challenges, hydrothermal liquefaction (HTL) has emerged as a promising thermochemical technique capable of converting complex organic waste into valuable bio-oil, offering a dual benefit of waste reduction and energy recovery [4,5,6,7]. Unlike pyrolysis-based technologies, HTL operates in a hot, compressed water environment, allowing efficient depolymerization of wet or heterogeneous feedstocks without the need for drying. Recent studies have demonstrated the feasibility of applying HTL to various biomass and composite wastes, including food residues, algae, and multilayered packaging materials [8,9]. However, despite these advances, the reaction kinetics of HTL applied to complex, heterogeneous, multiphase feedstocks such as Tetra Pak remain insufficiently explored.

In most HTL kinetic studies, the entire biomass feedstock is simplified into a lumped pseudo-component and modeled as a single first-order decomposition reaction that yields several lumped product phases such as biocrude, aqueous products, gas, and solid residues. Representative examples include the lumped kinetic model developed by Elliott and Schmidt, who treated lignocellulosic biomass as a single reactant decomposing into oil, gas, char, and water-soluble organics following first-order Arrhenius behavior [10]. Jena et al. applied similar single-lump first-order kinetics for algal HTL, assuming a global reaction converting biomass to biocrude and byproducts [11]. More recently, Akhtar and Amin also used a lumped model for municipal sludge HTL, where the feedstock was assumed to decompose via one-step first-order kinetics to yield oil, gas, and solid residues [12]. Qian et al. developed a global kinetic model correlating the yields of biocrude, aqueous products, solids, gases, and volatiles from sewage sludge under various severities [13], while Yadav et al. proposed a generalized reaction network for water hyacinth [14]. Although useful for process-level predictions, such models cannot capture the distinct decomposition behaviors, reaction pathways, and oil-forming contributions of different components within a heterogeneous composite. In the case of Tetra Pak, cellulose and LDPE follow fundamentally different hydrothermal degradation mechanisms, and aluminum does not participate chemically but influences heat and mass transfer. Without component-specific kinetic information, mechanistic understanding and reactor-scale optimization remain limited.

Although Tetra Pak is a low-moisture feedstock, the hydrothermal environment provides unique reaction pathways that cannot be achieved through pyrolysis or gasification. The cellulose-rich paper layer (≈75 wt%) undergoes rapid hydrolysis, dehydration, and depolymerization only in the presence of compressed water, which effectively suppresses char formation. Furthermore, the high-pressure aqueous phase promotes aluminum–polymer delamination and enhances the scission and hydrogen-donation reactions of LDPE, enabling the formation of liquid intermediates that do not occur in conventional pyrolysis. Therefore, the purpose of this study is not to claim that HTL is inherently superior for dry feedstocks but to establish a mechanistic and kinetic understanding of how each component of the Tetra Pak composite decomposes under hydrothermal conditions.

It should also be emphasized that the present work focuses on fundamental decomposition behavior and quantitative kinetic modeling, rather than process economics or industrial optimization. While the requirement of water addition may limit large-scale deployment, HTL remains an essential platform for elucidating the multiphase reaction network and component-specific decomposition pathways of complex composite wastes such as Tetra Pak.

In this study, the paper–plastic–aluminum composite waste was deconstructed into its primary components—polyethylene (PE), paper fibers, and aluminum. Based on experimental data, we established individual reaction network models for the conversion of each component into bio-oil, solid residues, and gases. The kinetic parameters of these models were subsequently optimized. And the comprehensive quantitative kinetics model was constructed to faithfully correlate the effects of reaction time and temperature on product yields. This integrated modeling approach provides new insights into the reaction mechanisms and optimization strategies for efficient bio-oil production from complex multi-component waste.

## 2. Materials and Experimental Methods

### 2.1. Materials and Equipment

The waste Tetra Pak was obtained from milk packaging cartons provided by Mengniu Co., Ltd. (Inner Mongolia, China). It was composed of 75 wt% paper, 20 wt% LDPE and 5 wt% aluminum. All mass fractions are expressed on a dry basis after drying the samples to constant weight. In the experiments, the packaging waste was cut into 1 × 1 mm fragments. The element composition was shown in Table 1.

For hydrothermal liquefaction (HTL) experiments, Tetra Pak was accurately weighed and mixed with deionized water, with the water amount determined based on the reactor volume, target temperature, and water density. The mixture was then loaded into a 20 mL microreactor. A tube furnace (GSL-1400X, Hefei Kejing Material Technology Co., Ltd., Hefei, China) was used for heating. The feed concentration was maintained at 20 wt%. The water density at the reaction temperature corresponded to a pressure of 20 MPa. Actually, the reaction pressure was not directly measured with a pressure gauge but was calculated. Specifically, the water amount was determined based on the density of water at the set reaction temperature and target pressure, together with the known effective reactor volume. Once the reactor was heated to the set temperature, the water density corresponded to the target autogenous pressure. Experiments were conducted at temperatures ranging from 320 to 440 °C and residence times between 10 and 50 min. The reaction time is defined as the duration for which the reactor is maintained at the set temperature after reaching it, excluding the heating period.

After the reaction, the reactor was thoroughly rinsed with 9 mL of ethyl acetate. The aqueous phase, solid phase, and ethyl acetate-containing oil phase were then centrifuged (H1850, Hunan Xiangyi Laboratory Co., Ltd., Changsha, China) at 9000 rpm for 20 min. Following phase separation, the oil phase was transferred to a test tube and purged under a nitrogen atmosphere using a nitrogen blower (JTN100-1, Hangzhou Jutong Electronics Co., Ltd., Hangzhou, China) at 30 °C to evaporate the ethyl acetate. When the mass change in the test tube stabilized within ±0.001 g, the bio-oil product was collected and weighed. The solid phase was dried in an oven at 80 °C for 8 h to obtain the solid residue.

### 2.2. Performance Characterization

The carbon (C) and hydrogen (H) contents of the bio-oil were measured using a VARIO EL Cube elemental analyzer (Elementar, Frankfurt, Germany), while the oxygen (O) content was estimated by calculation. Functional groups were identified using an FTIR-8400S spectrometer (Shimadzu, Kyoto, Japan) over a range of 500–4000 cm^−1^. Boiling point distribution was analyzed with thermogravimetric analysis (TGA) on a 209 F3 Tarsus system (Netzsch, Selby, Germany), using nitrogen gas (60 mL/min), heating from 25 °C to 550 °C at 10 °C/min.

### 2.3. Data Processing

The formula for calculating the bio-oil yield (*Y_bo_*) is as follows:

(1) $$Y_{bo}=\frac{M_{1}}{M_{0}}\times 100\%$$

The higher heating value (HHV) of each component was estimated using the modified Dulong formula [15], which is widely applied for biomass fuels, which is expressed as follows:

(2) HHV=0.338C+1.428(H−0.125O)

M_0_ is the mass of the raw material input (g); M_1_ is the mass of the produced bio-oil (g); and C, H, and O represent the mass fractions of carbon, hydrogen, and oxygen elements, respectively. All mass fractions reported for paper, polyethylene, and aluminum were determined on a dry mass basis. The Tetra Pak samples were oven-dried at 105 °C to constant weight prior to compositional analysis.

The kinetic parameters of the component-specific reaction networks were obtained by nonlinear regression using MATLAB R2023b with the lsqnonlin solver from the Optimization Toolbox. A trust-region-reflective algorithm was employed to minimize the squared error between experimental yields and model-predicted values.

The agreement between experimental data and model predictions was assessed using standard statistical metrics. For each product fraction, the coefficient of determination (R^2^) was calculated to quantify the proportion of variance explained by the kinetic model. In addition, residuals were computed as the difference between experimental and calculated yields and plotted as residual plots to evaluate systematic bias and variance assumptions.

## 3. Results and Discussions

### 3.1. Effect of Temperature and Time on the Bio-Oil Yield

**Figure 1:**
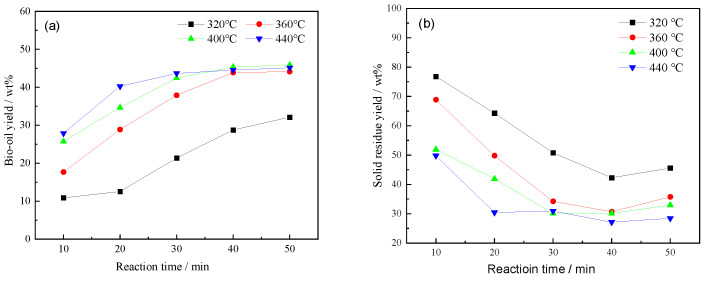
Effect of reaction temperature and retention time on yields of liquefaction products (**a**) bio-crude oil, (**b**) solid residue.

Figure 1 showed the variations in bio-oil and solid residue yields with reaction time at different temperatures (320–440 °C). The use of 440 °C in this study aimed to capture the high-temperature decomposition behavior of LDPE and to obtain reliable kinetic parameters over a broad range of severities. This temperature was not proposed as an optimal operating point but serves to identify the onset of PE depolymerization and secondary cracking. As illustrated in Figure 1a, the bio-oil yield increased continuously with time at all temperatures, reaching a plateau after approximately 40–50 min. The yield rose significantly with increasing temperature, indicating that higher thermal severity promotes depolymerization and secondary condensation reactions leading to oil formation.

Conversely, Figure 1b demonstrated that the solid residue yield decreased sharply during the initial reaction period and then stabilized, suggesting rapid conversion of solid components into liquid and gaseous products. The lowest solid yield and highest oil yield were both obtained at 400–440 °C, implying that this temperature range is optimal for maximizing bio-oil production while minimizing char formation.

Similar results have been reported in earlier studies. Dava et al. [16] found that moderate temperatures are best for bio-oil production, as they balance breakdown and recombination of molecules. In contrast, higher temperatures lead to more gas formation due to over-cracking. Dave et al. [16] also noted that too short a time results in incomplete breakdown of biomass, while very long times can cause secondary reactions that lower oil quality. Gong and Jiang [17] also noted that there was an optimal temperature range for hydrothermal liquefaction—beyond that, oil yield drops as more volatile gases are produced. Ni [18] emphasized that reaction time strongly affects the balance between oil, gas, and solids. Overall, the best oil yield is obtained at 360 °C after 50 min, when the system nears equilibrium.

### 3.2. Boiling Point Distribution of Bio-Crude

Table 2 showed how the boiling point distribution of bio-oil from Tetra Pak changes with reaction time during hydrothermal liquefaction (HTL). The boiling point distribution of the bio-oil was estimated from the derivative thermogravimetric curves according to standard thermogravimetric distillation methods [19]. In this approach, the temperature corresponding to each mass-loss event was treated as an equivalent boiling temperature of the corresponding pseudo-fraction. The mass fraction volatilized below 200 °C was assigned to gasoline-range compounds, 200–350 °C to diesel-range fractions, 350–500 °C to heavy oil fractions, and the remaining mass above 500 °C was attributed to non-volatile or residue components. Relative fraction percentages were calculated by dividing the mass loss occurring within each temperature interval by the total volatilized mass. This method assumes that thermogravimetric volatilization under an inert atmosphere mimics the atmospheric distillation behavior of complex bio-oil mixtures, a widely used approximation in TGA-based fuel characterization. Similar TGA-derived boiling point distribution analysis has been widely used in the characterization of pyrolysis oils and HTL biocrude [20,21].

The naphtha fraction (light compounds) was highest at 10 min (27.6%), suggesting early thermal cracking produces small, volatile molecules. However, its content drops later (e.g., 20.1% at 20 min), likely due to secondary reactions or gas loss [22]. The kerosene fraction remained fairly stable, with a slight increase at 40 min (26.2%). This suggested that medium-length hydrocarbons are consistently formed during the process.

The diesel fraction was the most abundant, peaking at 40 min (30.0%). This indicated that mid-sized molecules were favored as the reaction continued, due to ongoing breakdown and recombination of smaller chains. Similar behavior was noted by Haque et al., where diesel-range bio-oil fractions increased steadily with longer reaction durations until secondary degradation began [23]. The vacuum residue fraction reached its highest level at 20 min (26.3%) and then decreased. This suggested that heavy compounds form early in the reaction but later break down into lighter products through processes like cracking or water-gas shift reactions. A high amount of residue may also indicate incomplete breakdown or carbon buildup, as seen in similar studies using TGA [24,25].

### 3.3. Infrared Analysis of Bio-Oil

**Figure 2:**
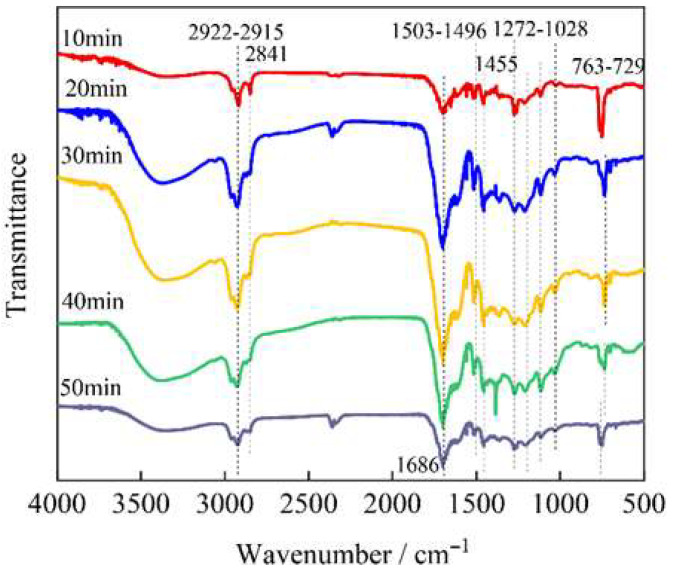
FT-IR spectra of bio-oil obtained at different reaction time at 360 °C.

FT-IR analysis (Figure 2) showed the chemical composition of bio-oil changes with reaction time at 360 °C. The absorption peaks at 2922–2841 cm^−1^, related to –CH_2_– groups, decrease over time, indicating reduced aliphatic content. Meanwhile, the peak at 1686 cm^−1^ strengthens, suggesting the formation of carbonyl compounds like ketones or aldehydes. Increased intensity in the aromatic regions (1503–1496 cm^−1^ and 763–729 cm^−1^) reflects growing aromatization and ring substitution. These trends matched the TGA results: as shown in Table 2, lighter fractions (naphtha and kerosene) stabilize or decline after 30 min, while heavier fractions (diesel and residue) increase, indicating more cracking and repolymerization. These findings aligned with previous studies [26,27] showing that longer hydrothermal treatment enhanced aromatization and heavy compound formation, affecting bio-oil quality.

## 4. HTL Model and Performance Analysis for Tetra Pak

### 4.1. Model Development

**Figure 3:**
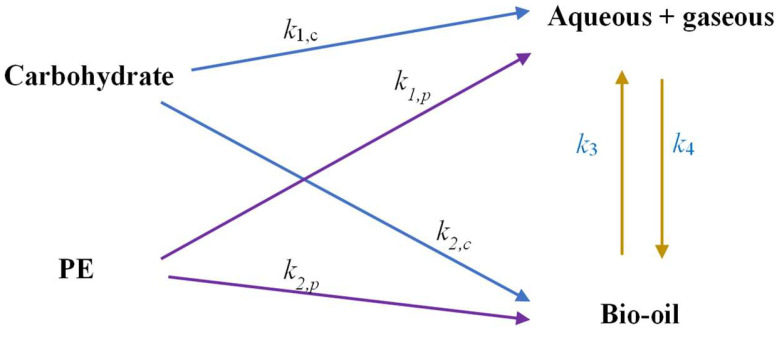
HTL reaction network incorporating biochemical content.

This study adopted a modified hydrothermal liquefaction (HTL) kinetics model originally proposed by Hietala et al. [28], incorporating the specific composition of paper–plastic–aluminum (Tetra Pak) waste. The reaction network (Figure 3) assumed that carbohydrates (paper), polyethylene (PE), and aluminum (Al) decomposed independently into aqueous/gaseous products (AP + GP), bio-oil, and solid residue through first-order reactions.

The mass balance for the solid fraction was given as follows:

(3) $$X_{1}\;=\;X_{1,C}\;+\;X_{1,P}\;+\;X_{1,A\;},$$

where subscripts C, P, and A denote carbohydrates, PE, and Al, respectively.

The conversion of each component follows first-order kinetics. For example, convsion of carbohydrates was described as follows:

(4) $$\frac{{\mathrm{dX}}_{1,c}}{\mathrm{dt}}=\;-\left(k_{1,c}\;+\;k_{2,c}\right)X_{1,c}\;,$$

Conversion of PE:

(5) $$\frac{{\mathrm{dX}}_{1,p}}{\mathrm{dt}}\;=\;\;-\left(k_{1,p}\;+\;k_{2,p}\right)X_{1,p}\;,$$

Yields of aqueous and gaseous (AP + GP):

(6) $$\frac{{\mathrm{dX}}_{2}}{\mathrm{dt}}\;=\;{k_{1,c}X}_{1,c}+k_{1,p}X_{1,p}+\;k_{3}X_{3}-k_{4}X_{3},$$

Yield of bio-oil:

(7) $$\frac{{\mathrm{dX}}_{3}}{\mathrm{dt}}\;=\;{k_{2,c}X}_{1,c}+k_{2,p}X_{1,p}-k_{3}X_{2}+k_{4}X_{2},$$

The yields of aqueous/gaseous products (X_2_) and bio-oil (X_3_) were expressed as functions of the primary decomposition and secondary conversion pathways.

Rate constants (*k_j_*) were obtained by fitting the model to experimental data using a least-squares optimization method, and Arrhenius parameters were subsequently determined by regression. Model performance was evaluated by the residual sum of squares between predicted and experimental yields. Figure 3 showed the reaction kinetics information for each pathway.

### 4.2. Model Correlation

Table 3 summarized the kinetic parameters for the hydrothermal liquefaction (HTL) of Tetra Pak, including the rate constants (*k*, min^−1^) determined at 320~440 °C and the apparent activation energies (*E*, kJ mol^−1^) for each reaction pathway. The carbonaceous fraction (Carb) showed much higher reactivity than polyethylene (PE), as reflected by its larger *k* values and lower activation energies (25.8–49.0 kJ mol^−1^). These results suggested that cellulose- and lignin-derived components readily decompose and form bio-oil under sub-/supercritical water conditions. In contrast, PE exhibited higher activation energies (54.9–78.3 kJ mol^−1^) and very low *k* values below 360 °C, indicating strong thermal stability and limited degradation until high temperatures are reached.

For the Carb fraction, the conversion to AP + GP showed the lowest activation energy (25.8 kJ mol^−1^), implying facile hydrolytic degradation and decarboxylation reactions. The formation of bio-oil from Carb (*E* = 49.0 kJ mol^−1^) proceeded via secondary condensation of oxygenated intermediates, representing the main oil-producing route at moderate temperatures. The intermediate transformation AP + GP → bio-oil (*E* = 30.1 kJ mol^−1^) further confirmed that soluble intermediates play a crucial role in oil formation through polymerization and dehydration pathways. Conversely, the reverse cracking reaction bio-oil → AP + GP (*E* = 55.5 kJ mol^−1^) became significant at higher temperatures, reducing oil yield and enhancing gaseous production.

Overall, the HTL process involved sequential decomposition, intermediate formation, oil generation, and thermal cracking. The optimal temperature range (360–400 °C) achieved a balance between bio-oil production and stability, with Carb as the dominant contributor and PE mainly enhancing oil hydrophobicity and heating value.

A comparison with pyrolysis and gasification was necessary for clarifying the applicability of HTL to Tetra Pak. Pyrolysis offered lower operating pressure and is suitable for dry materials; however, it typically resulted in higher solid char formation from cellulose and poor separation of the aluminum layer, which becomes embedded in carbonaceous residues [29,30]. In contrast, the hydrothermal environment suppresses excessive carbonization, generates higher liquid yields from the paper fraction, and facilitates physical separation of aluminum [16]. More importantly, the formation and transformation of aqueous intermediates (AP + GP → oil) represented unique pathways that are essential to construct the kinetic network developed in this work but cannot be observed in pyrolysis systems.

Table 4 compares the kinetic parameters obtained in this study with those reported for various biomass and biopolymer feedstocks. The rate constants of Tetra Pak hydrothermal liquefaction were generally lower than those of microalgae (e.g., *Chlorella vulgaris*, *Scenedesmus* sp.) and lignocellulosic materials, indicating slower decomposition under similar conditions. The activation energies (25.8–55.5 kJ mol^−1^) of Tetra Pak were moderate compared with those of pure polymers or microalgae, suggesting that the composite nature of the feed leads to a more complex reaction network involving both carbohydrate and polyethylene fractions. The kinetic behavior of Tetra Pak waste was slower than that of easily degradable biomasses such as microalgae and cellulose, reflecting its composite nature containing both carbohydrate and plastic fractions.

**Figure 4:**
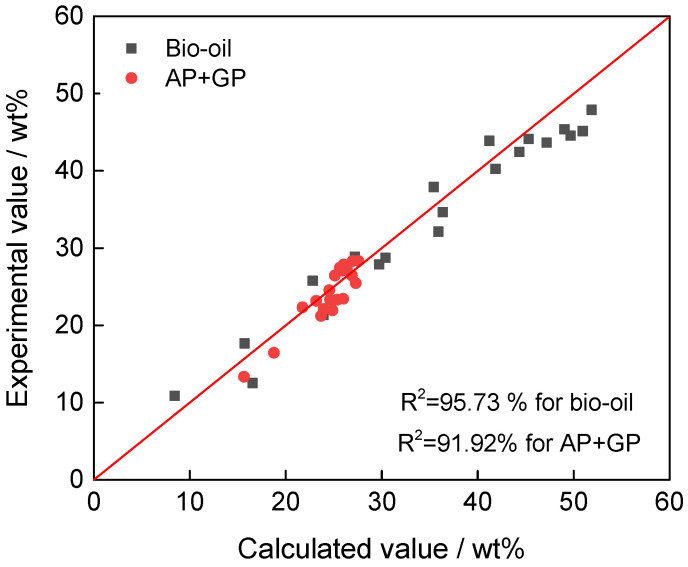
Experimental (discrete points) and calculated (continuous curves) products yields from HTL at 320 °C 360 °C and 400 °C and 440 °C.

**Figure 5:**
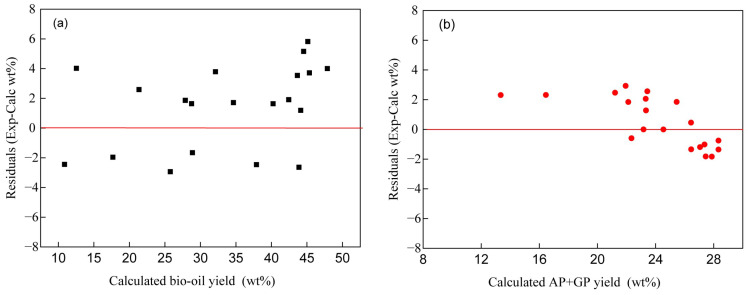
Residual plot of the kinetic model for bio-oil (**a**) and AP + GP yield (**b**).

Figure 4 and Figure 5 show the comparison between experimental and model-predicted product yields for bio-oil (black squares) and AP + GP (red circles), along with the corresponding residual plots. The data points closely follow the diagonal line, with coefficients of determination of 95.73% for bio-oil and 91.92% for AP + GP, indicating strong agreement between experimental measurements and the kinetic model. Residuals are randomly scattered around zero for both products, showing no systematic trend and confirming the adequacy of the first-order lumped kinetic model.

### 4.3. Analysis of Bio-Oil Generation Rates

**Figure 6:**
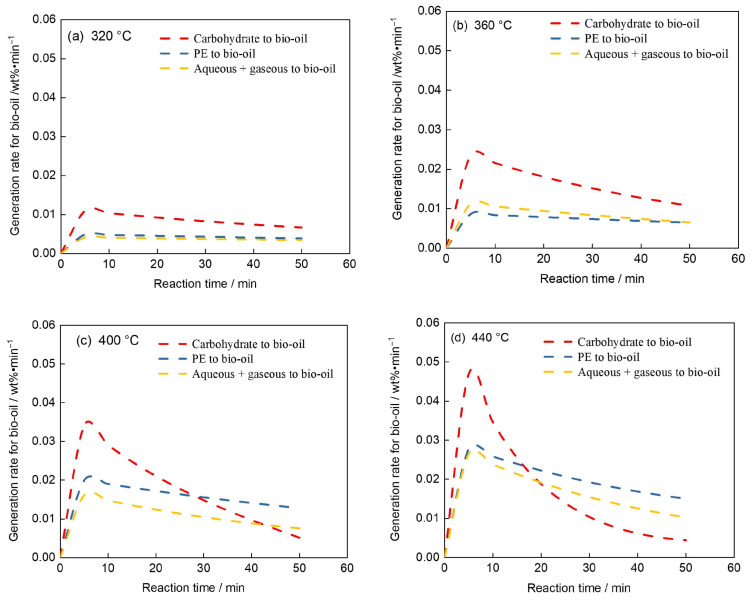
Bio-oil generation rate analysis. ((**a**) 320 °C, (**b**) 360 °C, (**c**) 400 °C, (**d**) 440 °C).

Figure 6 illustrated the variation in bio-oil generation rates with reaction time at different temperatures (320–440 °C). At all temperatures, the bio-oil formation rate initially increases rapidly, reaches a maximum within the first 10–15 min, and then gradually decreases, indicating that bio-oil was mainly produced during the early stage of hydrothermal liquefaction (HTL). The peak generation rate rose markedly with increasing temperature, suggesting enhanced decomposition and secondary conversion of intermediates. Among all pathways, the contribution from carbohydrate decomposition was dominant, while polyethylene and aqueous/gaseous intermediates showed lower but sustained rates at higher temperatures. At 440 °C, the rapid decline in rate after the peak implies intensified secondary cracking of bio-oil into lighter products. These trends confirmed that temperature strongly promotes both oil formation and degradation, with optimal oil generation occurring around 360–400 °C.

## 5. Conclusions

Hydrothermal liquefaction (HTL) of paper–plastic–aluminum (Tetra Pak) waste was systematically investigated in the temperature range of 320–440 °C and residence times of 10–50 min. The results show that bio-oil yield increased with both temperature and time, reaching a maximum of 43 wt% at 360 °C and 50 min, while solid residue decreased correspondingly. Boiling point analysis revealed that the diesel-range fraction dominated the bio-oil, indicating effective conversion of macromolecular components into mid-chain hydrocarbons. FT-IR spectra confirmed a progressive increase in carbonyl and aromatic structures with longer reaction time, implying enhanced secondary condensation and aromatization.

Kinetic modeling based on first-order reactions accurately reproduced the experimental yields, yielding activation energies between 25.8 and 78.3 kJ mol^−1^. Carbohydrates exhibited higher reactivity than polyethylene, contributing mainly to bio-oil formation through hydrolysis and dehydration, while PE decomposition became significant only above 360 °C.

This study provides a comprehensive understanding of the thermal–kinetic behavior of composite packaging waste under hydrothermal conditions, supporting the development of efficient waste valorization technologies for sustainable fuel production. The limitation regarding water addition and energy input has been explicitly acknowledged. Industrial hydrothermal liquefaction processes typically operate with closed-loop water recycling systems that reuse more than 90–95% of process water. While such engineering optimization is beyond the scope of this kinetic study, these considerations have now been discussed as important aspects for future scale-up.

## Figures and Tables

**Table 1:** Element composition of Tetra Pak.

Element	Composition (wt%)
C	50.55
H	6.226
O^a^	38.07
HHV (MJ/kg)	19.18

O^a^ was calculated by difference. The remaining ~5 wt% corresponds to the aluminum foil layer and minor inorganic ash in the Tetra Pak composite.

**Table 2:** Boiling point distribution of bio-crude samples based on cut points for crude oil distillate fractions.

Fraction	Boiling Point Range	Carbon Number Range	Relative Content (%)			
			10 min	20 min	30 min	40 min
Naphtha	25–200 °C	C7–C10	27.6	20.1	26.5	21.9
Kerosene	200–300 °C	C11–C15	32.9	24.5	25.5	26.2
Diesel	300–550 °C	C16–C47	28.3	29.0	27.7	30.0
Vacuum Oil Residue	≥550 °C	≥C48	11.3	26.3	20.4	22.0

**Table 3:** Optimized rate constants at different reaction temperature.

Subscript	Reaction	*k* (min^−1^)				*E* (KJ/mol)
		320 °C	360 °C	400 °C	440 °C	
1,C	Carb → AP + GP	0.0328	0.045	0.06	0.0794	25.78
1,P	PE → AP + GP	0	0.00168	0.00543	0.00918	78.27
2,C	Carb → bio-oil	0.0116	0.0257	0.04	0.0637	49.01
2,P	PE → bio-oil	0.005	0.00891	0.0209	0.03005	54.91
3	AP + GP → bio-oil	0.0203	0.0312	0.0435	0.0569	30.12
4	bio-oil → AP + GP	0.00421	0.0118	0.0176	0.0296	55.47

**Table 4:** Rate constant (*k*_j_, min^−1^) at each temperature (°C) for different feed.

Feed	Reaction Rate Constant *k*/min^−1^			*E* (KJ/mol)	Ref.
	320	360	400		
Carb → AP + GP					
Tetra Pak	0.0328	0.045	0.06	25.78	This paper
Spirulina	0.1234	0.1207	0.1382	9.87	[31]
Cellulose	0.27	--	--	68.92	[32]
Xylan	0.37	--	--	2.88	[32]
Alkali lignin	0.4	--	--	2.88	[32]
*Aurantiochytrium* sp.	0.0331	0.0506	0.0724	33.08972	[28]
*C. protothecoides*	0.3234	0.4649	0.6333	27.89347	[28]
*Scenedesmus* sp.	0.3613	0.5259	0.7239	28.84958	[28]
*Chlorella vulgaris*	0.9788	1.1048	--	9.4521866	[28]
Carb→bio-oil					
Tetra Pak	0.0116	0.0257	0.04	49.01	This paper
Spirulina	0.0224	0.008	0.0788	11.15	[31]
Cellulose	0.16	--	--	74.29	[32]
Xylan	0.14	--	--	25.74	[32]
Alkali lignin	0.24	--	--	64.83	[32]
*Aurantiochytrium* sp.	0.0624	0.1378	0.2705	60.88	[28]
*C. protothecoides*	0.0013	0.0049	0.0155	102.86	[28]
*Scenedesmus* sp.	0.0004	0.0021	0.0076	122.34	[28]
*Chlorella vulgaris*	0.0004	0.0008		54.10	[28]
AP + GP→bio-oil					
Tetra Pak	0.0203	0.0312	0.0435	30.12	This paper
Spirulina	0.2965	0.3022	0.2447	10.12	[31]
Celluslose	0.13	--	--	38.32	[32]
Xylan	0.007	--	--	20.52	[32]
Alkali lignin	0.13	--	--	28.06	[32]
*Aurantiochytrium* sp.	0.0895	0.1498	0.2324	39.74	[28]
*C. protothecoides*	0.0057	0.013	0.0262	63.52	[28]
*Scenedesmus* sp.	0.004	0.0078	0.0137	51.27	[28]
*Chlorella vulgaris*	0.407	0.4867	--	14.00	[28]
Bio-oil→AP + GP					
Tetra Pak	0.00421	0.0118	0.0176	55.47	This paper
Spirulina	0.2841	0.3482	0.2977	10.39	[31]
Celluslose	0.12	--	--	43.93	[32]
Xylan	0.038	--	--	23.93	[32]
Alkali lignin	0.13	--	--	28.16	[32]
*Aurantiochytrium* sp.	0.0848	0.1346	0.1995	35.51	[28]
*C. protothecoides*	0.0039	0.0129	0.0352	91.34	[28]
*Scenedesmus* sp.	0.0035	0.0114	0.031	90.54	[28]
*Chlorella vulgaris*	0.3233	0.4072	--	18.01	[28]

## Data Availability

Data is contained within the article.
